# Robust Dehaze Algorithm for Degraded Image of CMOS Image Sensors

**DOI:** 10.3390/s17102175

**Published:** 2017-09-22

**Authors:** Chen Qu, Du-Yan Bi, Ping Sui, Ai-Nong Chao, Yun-Fei Wang

**Affiliations:** 1 College of Aeronautics and Astronautics Engineering, Air Force Engineering University, Xi’an 710038, China; biduyan@126.com (D.-Y.B.); chaoainong@163.com (A.-N.C.); wyfpost@163.com (Y.-F.W.); 2 Foundation Department, Air Force Engineering University, Xi’an 710051, China; 3 Information and Navigation College, Air Force Engineering University, Xi’an 710077, China; ziwuningxin@163.com

**Keywords:** CMOS image sensors, image dehaze, atmospheric scattering model, local consistent Markov random field

## Abstract

The CMOS (Complementary Metal-Oxide-Semiconductor) is a new type of solid image sensor device widely used in object tracking, object recognition, intelligent navigation fields, and so on. However, images captured by outdoor CMOS sensor devices are usually affected by suspended atmospheric particles (such as haze), causing a reduction in image contrast, color distortion problems, and so on. In view of this, we propose a novel dehazing approach based on a local consistent Markov random field (MRF) framework. The neighboring clique in traditional MRF is extended to the non-neighboring clique, which is defined on local consistent blocks based on two clues, where both the atmospheric light and transmission map satisfy the character of local consistency. In this framework, our model can strengthen the restriction of the whole image while incorporating more sophisticated statistical priors, resulting in more expressive power of modeling, thus, solving inadequate detail recovery effectively and alleviating color distortion. Moreover, the local consistent MRF framework can obtain details while maintaining better results for dehazing, which effectively improves the image quality captured by the CMOS image sensor. Experimental results verified that the method proposed has the combined advantages of detail recovery and color preservation.

## 1. Introduction

**Figure 1:**
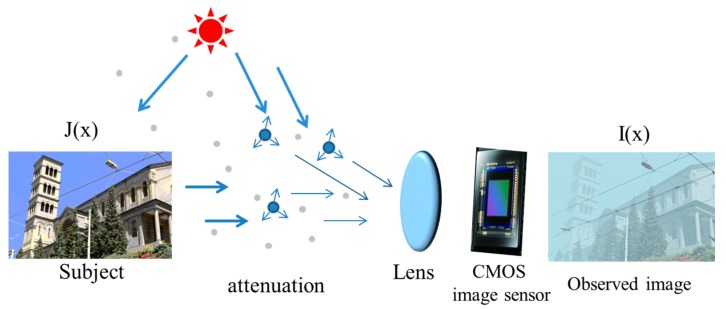
Image degradation model of the Complementary Metal-Oxide-Semiconductor (CMOS) image sensor.

Complementary Metal-Oxide-Semiconductor (CMOS) [1] image sensors, given their high integration, low power consumption, small size, low cost and other advantages, have been widely used in environmental monitoring, intelligent navigation [2], outdoor tracking, and so on. The outdoor collection of visible light images will inevitably encounter cloudy weather (especially in recent years, haze weather), resulting in the reduction of visibility, contrast and sharpness of the scene, therefore making monitors, intelligent navigation, and outdoor object recognition difficult. A haze image is generated as shown in Figure 1.

As one of the most commonly used probabilistic graphical models, the Markov random field (MRF) provides not only an effective framework for modeling the statistical prior of natural images [3,4], but also a means of making inferences about images [5]. These inferences, widely investigated and applied to solve problems such as image reconstruction [6], segmentation [7], denoising [8,9,10], inpainting [11], concern underlying image and scene structure. In a CMOS for a single outdoor image, MRF has been informally used to model the haze scene and improve the dehaze result in some works. Fattal [12] assumed that the surface Lambertian shading factor and the scene transmission were locally independent to separate the haze from the scene, and then used a Gaussian MRF to smooth the transmission values. This was physically valid, but the assumption was too strong for a variety of images, and thus tended to under-estimate the haze thickness in practice. Based on the two observations that (1) images with enhanced visibility have more contrast than hazy images, and (2) air-light tends to be smooth, an interesting single image haze removal algorithm was proposed by maximizing the local contrast of the restored image in the framework of MRF in Reference [13]. The results were visually compelling; however, the method might not be physically valid. Nishino [14] introduced a novel Bayesian probabilistic method that modeled the image with a factorial MRF where the scene albedo and depth were two statistically independent latent layers and jointly estimated. Most details could be recovered from the haze image by this approach, but the result often suffered from oversaturation. Caraffa [15] proposed a novel MRF model for the single image dehazing problem, which could easily be refined to obtain better results on road images using the planar constraint; however, this method only outperformed the state-of-the-art in single road scene image dehazing.

As above-mentioned, these MRF-based techniques focus on traditional first-order MRF to model a single hazy image in References [12,13,14,15]. However, in a CMOS, the traditional first-order MRF only models the statistical dependency between the neighboring pixels and constrains image on the local prior information, not the whole image. To better capture the image structures, higher-order MRF extends its models to larger neighborhoods. Compared with first-order MRF, higher-order MRF incorporates more sophisticated qualitative and statistical priors, resulting in stronger modeling power. However, it is harder to minimize their energy functions and it is much more complex to estimate their parameters due to the explosive growth of their number. Therefore, higher-order MRF applications in image processing are still seldom used for the limitation of energy function optimization algorithm.

To enhance the first-order MRF constraint ability of the whole image and avoid too much complexity by the higher-order MRF algorithm, we propose a novel MRF dehazing method based on local consistent patches. The proposed method is unlike the traditional first-order MRF, which requires second-order clique potentials to constrain the neighboring relationships, and nor like the higher-order MRF that needs higher-order potentials defined on the overlapping cliques. There are two basic observations: the depth change of a hazy image is usually gradual, and the correct depth values in neighbor tend to be the same, hence, regardless of their scattering coefficient, the medium transmission map can be considered as a constant in a small patch; atmospheric light, tending to be smooth, mainly depends on the distance of scene and can be roughly considered as a constant in local. Relying on these two observations, we developed a linear model at small patches between the hazy image and the restored image, and then proposed a cost function in local consistent MRF. Our major contribution in this paper is that a local consistent range MRF was constructed by extending the neighboring range clique around the pixels to a non-neighboring range clique, where several similar patches in an effective window exist around each pixel. In this framework, both of the neighboring pixel structures and the correlations among the similar patches can be captured. This local consistent MRF cost function can be efficiently optimized by gradient-based methods. Thus, as our processing is carried out within the effective windows around each pixel, it can be considered as a local filter for a CMOS image sensor.

Very recently, He [16] developed a dehazing method based on local filtering which used a neighboring window-based operation and a local linear model, namely, soft matting. However, this method has an obvious limitation, that is, it may exhibit halo artifacts near some edges. To address this problem, many edge-aware weights were incorporated into the local-based filtering. Interestingly, by explicitly simplifying our cost function with respect to model parameters, we found that the cost function constructed by local consistent MRF had a dramatic relationship with the soft matting. Furthermore, unlike the soft matting, our method had an additional edge-preserving term. So, compared with the soft-matting, the proposed local consistent MRF model avoided the halo artifacts and restored more detailed information. Experimental results also verified its effectiveness.

The rest of this paper is organized as follows. In Section 2, we present the image degradation model due to the presence of haze in the scene, and in Section 3 we construct the local consistent MRF to better restrain the whole image. Considering that both the atmospheric light and medium transmission map satisfied local consistency, we solve the local consistent MRF in Section 4 and report the experiment results in Section 5. In Section 6, we summarize the proposed approach and discuss its limitations.

## 2. Degradation Model

During inclement weather conditions such as fog, haze, and mist, in images captured by CMOS sensor devices light passing through the scattering medium is attenuated along its original course and distributed to other conditions. This process is commonly modeled mathematically by an atmospheric scattering model, which is widely used in computer vision and image processing [17,18,19,20], and can be expressed as follows:

(1) I(x)=J(x)t(x)+A(x)(1−t(x))

The first term is the direct attenuation and the second term is the air-light. *I*(*x*) is the image capture from the CMOS sensor device; *x* is the 2D spatial location; and *J*(*x*) is the real scene to be recovered. *A*(*x*) is the atmospheric light, which describes the ambient light in the scene. Existing methods commonly assume that *A*(*x*) is globally constant and it is independent from location *x*; however, in practice, the variation of the values of *A*(*x*) is dependent on the scene depth, and cannot be deemed as a constant in a nutshell. *t*(*x*) is the medium transmission map which describes the portion of the light that is not scattered and reaches the camera:

(2) $$t\left(x\right)=e^{-\beta \left(x\right)d\left(x\right)}$$

where *β*(*x*) is the scattering coefficient and is regarded as a constant in a homogeneous atmosphere condition; and *d(x)* is the distance from the scene point to the camera. The aim of haze removal is to restore the haze-free image *J*(*x*) from the hazy image *I*(*x*). It is a challenging problem as haze is dependent on the unknown depth information *d*(*x*) as seen in Equation (2). In addition, it is ill-posed as the input is only a single hazy image while the components *t*(*x*), *A*(*x*) and *J*(*x*) are all unknown. To restore the haze-free image *J*(*x*), both the atmospheric light *A*(*x*) and the medium transmission map *t*(*x*) need to be estimated. Once *A*(*x*) and *t*(*x*) are estimated, the dehazed image *J*(*x*) is obtained by:

(3) $$J\left(x\right)=\frac{1}{t\left(x\right)}\left(I\left(x\right)-A\left(x\right)\right)+A\left(x\right)$$

where 0 < *t*(*x*) ≤ 1, and we define *a_x_* as the 1/*t*(*x*) and *c_x_* as the *A*(*x*). Therefore, in this paper, the atmospheric scattering model can be simplified as:

(4) $$J\left(x\right)=a_{x}\left(I\left(x\right)-c_{x}\right)+c_{x}$$

## 3. Local Consistent Markov Random Fields

The energy functions for many commonly used MRF models can be written as a sum of unary and pairwise cliques [21,22]:

(5) $$E\left(x\right)=\sum_{m\in v}\phi_{m}\left(x_{m}\right)+\sum_{\left(m,n\in \varepsilon \right)}\psi_{m,n}\left(x_{m},x_{n}\right)$$

where *ν* corresponds to the location set of all image pixels, and *ε* is the set of all neighboring pixels of *m*. The neighboring set is commonly chosen to be 4-neighborhood. The random variable *x_m_* denotes the configuration of pixel m of the image. Every possible assignment of the random variable *x* defines a restored image. The unary potential $\phi_{m}$ is defined as the cost of a label being assigned to pixel *m* and can be computed using sophisticated potential functions such as color, texture, location, shape prior, and so on. The pairwise terms *ψ*_*m*,*n*_, mostly considered as smooth terms, are typically defined as an edge feature based on the difference between neighboring pixels. The use of the pairwise terms in the MRF model makes it favor smooth object boundaries. Bearing the risk of undesirable side effects, it improves results in most cases, and restored images obtained by pairwise terms tend to be over-smooth and often do not extract the fine details of the scene. Additionally, pairwise terms modeling the image prior statistics in small image patches do not easily generalize to priors for entire images [23]. This limits their impact in machine vision applications.

To address the over-smooth problem and use this formulation to model the statistics of entire images [24,25], a higher-order method has been proposed. Unlike the conventional MRF model explained in the previous section, the clique potentials of a higher-order Markov random field model are extended by incorporating higher-order terms defined on sets or regions of pixels [3,26,27]. The cost function of this higher-order MRF model can be written as:

(6) $$E\left(x\right)=\sum_{m\in v}\phi_{m}\left(x_{m}\right)+\sum_{\left(m,n\in \varepsilon \right)}\psi_{m,n}\left(x_{m},x_{n}\right)+\sum_{c\in S}\phi_{c}\left(x_{c}\right)$$

where *S* refers to a set of image regions defined on super-pixels, and *φ_c_* are higher-order terms defined on them. The framework described above is quite flexible and can be used to extract more detail from the restored images. However, the complexity of the algorithm for optimizing the cost function increases linearly with the size of the clique. This hinders wide use of the higher-order MRF model in image processing.

### 3.1. Basic Definition

**Figure 2:**
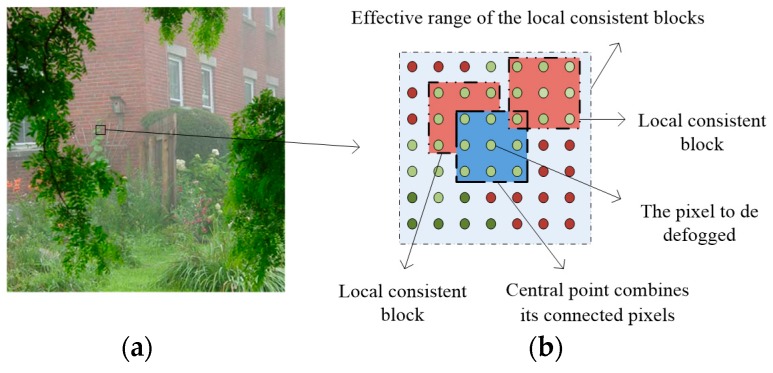
Local consistent Markov random field (MRF). (**a**) The input image; and (**b**) The local consistent blocks.

Images degraded by haze are normally taken from outdoor natural scenes, therefore, the change in scene depth is usually gradual and the correct depth values generally satisfy the local smooth feature, except for pixels at depth discontinuities, whose number is relatively small. At the same time, we found that within the local pixel block, the same colors had the same scene depth changes. This discovery enabled us to use the image color feature to find the local consistent blocks about the scene depth information. As shown in Figure 2, we defined a local consistent MRF model based on these local consistent blocks.

Unlike the conventional cliques of MRF that are generally defined by the relationship between the neighboring pixels or the super-pixels, we designed the local consistent range of cliques which captured both the neighboring pixels’ structures and the correlations among the local similar patches. To take advantage of local consistent MRF in image dehazing, we first defined the local consistent blocks; and color moment is a kind of simple and effective representation in color feature. Thus, we used the second-order color moment to measure the similarity of the different image blocks in this paper.

In local consistent MRF, the clique of each pixel is composed of the neighboring pixels around the pixel and its top similar blocks, searched by block matching with squared error in all color channels as block similarity in an effective neighborhood around the pixel. As shown in Figure 2, the clique of pixel *m* (shown in the central point) combined its connected pixels (shown in the blue block) and the similar blocks (shown in the red blocks) in an effective neighborhood. Apparently, the local consistent blocks of pixel were adaptively located over the effective regions, and we selected the top six blocks by similarity as the local consistent blocks according to the squared errors.

**Figure 3:**
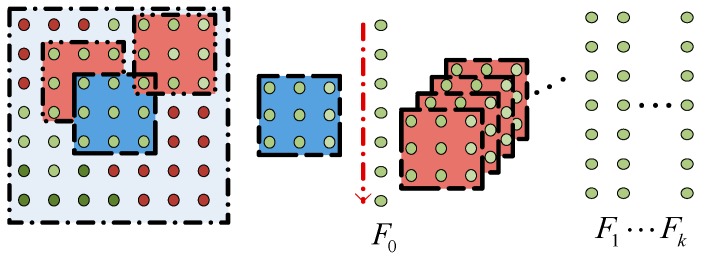
Vector representation of clique pixels.

Based on the above definitions, we denoted the clique of pixel m as *F*(*x_m_*), combined with two separations: 1-dimensional vector *F*_0_(*x_m_*) and *F_k_*(*x_m_*). *F*_0_(*x_m_*) was defined over pixel and its neighboring pixels called the neighboring block. *F_k_*(*x_m_*) was defined across all the top *k* similarity blocks of the pixel, as shown in Figure 3. The variable *x_m_* denoted the labeling of pixel m of the image. Then, *F*(*x_m_*) can be written as:

(7) $$F\left(x_{m}\right)=\left\{F_{0}\left(x_{m}\right),F_{1}\left(x_{m}\right),\cdots ,F_{k}\left(x_{m}\right)\right\}$$

where the similarity between the neighboring block *F*_0_(*x_m_*) and the local consistent blocks *F_k_*(*x_m_*) can be defined by squared errors, which can be denoted by:

(8) $$\sigma_{k}^{2}\left(x_{m}\right)=\sum_{i=1}^{3}\left(F_{0}\left(x_{m}^{i}\right)-F_{k}\left(x_{m}^{i}\right)\right){\left(F_{0}\left(x_{m}^{i}\right)-F_{k}\left(x_{m}^{i}\right)\right)}^{T}$$

where $x_{m}^{i}$ is the color label of pixel *m* in channel i,i∈{r,g,b}.

### 3.2. Formulation of Local Consistent MRF Model

We used cliques composed of neighboring pixels around the pixel and its top similar blocks to develop the clique potentials instead of the pairwise and higher-order cliques as this method not only strengthened the constraint of conventional MRF models, but also avoided the complexity. Meanwhile, the cost function of local consistent MRF can be represented as:

(9) $$E\left(x\right)=\sum_{m\in n}\phi_{m}\left(x_{m}\right)+\sum_{m\in n}\sum_{k}\phi \left(F_{0}\left(x_{m}\right),F_{k}\left(x_{m}\right)\right)$$

where the first term $\sum_{m\in \nu }\phi_{m}\left(x_{m}\right)={\left|x_{m}-{\tilde{x}}_{m}\right|}^{2}$ was used to constrain the relationship between the correct value $x_{m}$; and the estimated value ${\tilde{x}}_{m}$ in pixel *m* to ensure that the error was as small as possible. The second term $\sum_{k}\phi \left(F_{0}\left(x_{m}\right),F_{k}\left(x_{m}\right)\right)=\sum_{k}\lambda {\left\|F_{0}\left(x_{m}\right)-F_{k}\left(x_{m}\right)\right\|}^{2}$ was used to describe the difference between the neighboring block and the local consistent blocks in pixel *m*. λ is a regularization parameter penalizing the difference.

## 4. The Solution of Local Consistent MRF

The problem described in Section 2 is a totally ill-posed problem; however, there are some clues or observations that can be considered as haze priors to resolve the ambiguity:

1. The input images degraded by haze are normally taken from outdoor natural scenes. Therefore, the scene depth change is usually gradual and the correct depth values of neighboring pixels tend to the same and, hence, the medium transmission map *t*(*x*) can be considered as a constant in a small patch, regardless of their scattering coefficient *β*(*x*).
2. The value variations of *A*(*x*) are dependent on the scene depth, that is, objects with the same depth have the same values of *A*(*x*). So, the values of *A*(*x*) tend to be the same in local except for the pixels at depth discontinuities, whose number is relatively small.

From the observations mentioned above, we can consider that atmospheric light *A*(*x*) and medium transmission map *t*(*x*) both satisfy the character of local consistency.

### 4.1. Construction of Local Consistent MRF Model

According to the clues above, the changes of *A*(*x*) and *t*(*x*) across the image tended to be smooth for the major pixels in local. Thus, atmospheric light and transmission map were assumed to be constants in one local patch. The image degradation model in Equation (4) was simplified as:

(10) $$\begin{array}{cccc} J\left(x\right) & =a_{x^{\prime }}\left(I\left(x\right)-c_{x^{\prime }}\right)+c_{x^{\prime }}, & & \\ & \;b_{x^{\prime }}=c_{x^{\prime }}-c_{x^{\prime }}a_{x^{\prime }}, & & \forall x\in p_{x^{\prime }}\\ & J\left(x\right)=a_{x^{\prime }}I\left(x\right)+b_{x^{\prime }} & & \end{array}$$

which was assumed to have constant values of $a_{x^{\prime }}$, $b_{x^{\prime }}$, and $c_{x^{\prime }}$ in one small local patch $p_{x^{\prime }}$ centered at $x^{\prime }$. As such, Equation (10) builds up a linear relationship between the transmission map and the input image.

Motivated by the above descriptions, we used the haze-free image *J*(*x*) to develop a local consistent MRF model for the estimation of transmission map and atmospheric light. We calculated the potential function of MRF as:

(11) $$E\left(\left\{a_{x^{\prime }},b_{x^{\prime }}\right\}|p_{x^{\prime }}\right)=E\left(\left\{J\left(x\right)\right\}|p_{x^{\prime }}\right)=\sum_{x}\phi_{x}\left(p_{x^{\prime }}|\left\{J\left(x\right)\right\}\right)+\lambda \sum_{x}\sum_{k}\phi \left(F_{0}\left(J_{x}\right),F_{k}\left(J_{x}\right)\right)$$

where $p_{x^{\prime }}$ is a small patch centered at *x′*; λ is a regularization parameter penalizing the smoothness term; and *k* represents the number of consistent neighboring patches selected. We defined the first term, the data term, as:

(12) $$\phi_{x}\left(p_{x^{\prime }}|\left\{J\left(x\right)\right\}\right)={\left\|a_{x^{\prime }}I\left(x\right)+b_{x^{\prime }}-\tilde{J}\left(x\right)\right\|}^{2}$$

where $\tilde{J}\left(x\right)$ is the initial value of the haze-free image J(x); and $a_{x^{\prime }}$ are two constants in local patch $p_{x^{\prime }}$. The second term, the smoothness term, was defined as:

(13) $$\begin{array}{cc} & \sum_{k}\phi \left(F_{0}\left(J_{x}\right),F_{k}\left(J_{x}\right)\right)=\sum_{k=1}^{6}{\left\|F_{0}\left(J_{x}\right)-F_{k}\left(J_{x}\right)\right\|}^{2}\\ = & \sum_{k=1}^{6}\sum_{i=1}^{3}\left[\left(a_{x^{\prime }}F_{0}^{i}\left(I_{x}\right)+b_{x^{\prime }}\right)-\left(a_{x^{\prime }}F_{k}^{i}\left(I_{x}\right)+b_{x^{\prime }}\right)\right]{\left[\left(a_{x^{\prime }}F_{0}^{i}\left(I_{x}\right)+b_{x^{\prime }}\right)-\left(a_{x^{\prime }}F_{k}^{i}\left(I_{x}\right)+b_{x^{\prime }}\right)\right]}^{T} \end{array}$$

In one local patch $p_{x^{\prime }}$, the cost function $E\left(a_{x^{\prime }},b_{x^{\prime }}\right)$ was equivalent to:

(14) $$\begin{array}{l} E\left(a_{x^{\prime }},b_{x^{\prime }}\right)=\sum_{x\in p_{x^{\prime }}}({\left(a_{x^{\prime }}I\left(x\right)+b_{x^{\prime }}-\tilde{J}\left(x\right)\right)}^{2}+\\ \;\lambda \sum_{k=1}^{6}\sum_{i=1}^{3}\left[\left(a_{x^{\prime }}F_{0}^{i}\left(I_{x}\right)+b_{x^{\prime }}\right)-\left(a_{x^{\prime }}F_{k}^{i}\left(I_{x}\right)+b_{x^{\prime }}\right)\right]{\left[\left(a_{x^{\prime }}F_{0}^{i}\left(I_{x}\right)+b_{x^{\prime }}\right)-\left(a_{x^{\prime }}F_{k}^{i}\left(I_{x}\right)+b_{x^{\prime }}\right)\right]}^{T})\\ \;=\sum_{x\in p_{x^{\prime }}}{\left\|a_{x^{\prime }}I\left(x\right)+b_{x^{\prime }}-\tilde{J}\left(x\right)\right\|}^{2}+N\lambda a_{x^{\prime }}^{2}\sum_{k=1}^{6}\sigma_{k}^{2}\left(I_{x}\right) \end{array}$$

where *N* is the pixel number in local patch $p_{x^{\prime }}$. We denoted $\Gamma_{x^{\prime }}\left(x\right)=\frac{1}{\sum_{k=1}^{6}\left(\sigma_{k}^{2}\left(I_{x}\right)+\varepsilon \right)}$, *ε* as a small constant and its value is 0.001. Then, Equation (14) can be derived as:

(15) $$E\left(a_{x^{\prime }},b_{x^{\prime }}\right)=\sum_{x\in p_{x^{\prime }}}{\left\|a_{x^{\prime }}I\left(x\right)+b_{x^{\prime }}-\tilde{J}\left(x\right)\right\|}^{2}+\frac{N\lambda }{\Gamma_{x^{\prime }}\left(x\right)}a_{x^{\prime }}^{2}$$

Recent work in He [16] referred to a similar cost function as “soft matting”. It is important to note the difference in our approaches. Soft matting is used as a local filter to refine the transmission map. Interestingly, with Equation (15), our cost function based on local consistent MRF had a dramatic relationship with soft matting. In other words, with the local consistent MRF model, we established the dehazing relationship between the method based on random field and the method based on local filter, such as soft matting. However, unlike soft matting, our method has an edge-preserving term and larger weights are assigned to pixels at edges than to pixels in flat areas. Due to the edge-preserving term, the local consistent MRF model could avoid halo artifacts and recover most details from the haze image.

To obtain the variables $a_{x^{\prime }},b_{x^{\prime }}$ accurately, Equation (15) can be expressed as follows:

(16) $$\left(a_{x^{\prime }},b_{x^{\prime }}\right)=argminE\left(a_{x^{\prime }},b_{x^{\prime }}\right)$$

To solve this, we first calculated the partial derivative of $E\left(a_{x^{\prime }},b_{x^{\prime }}\right)$ and made them equal to zero:

(17) $$\frac{\partial E\left(a_{x^{\prime }},b_{x^{\prime }}\right)}{\partial a_{x^{\prime }}}=2\sum_{x\in p_{x^{\prime }}}(\left(a_{x^{\prime }}I\left(x\right)+b_{x^{\prime }}-\tilde{J}\left(x\right)\right)\cdot I\left(x\right))+\frac{2N\lambda }{\Gamma_{x^{\prime }}\left(x\right)}a_{x^{\prime }}=0$$

(18) $$\frac{\partial E\left(a_{x^{\prime }},b_{x^{\prime }}\right)}{\partial b_{x^{\prime }}}=2\sum_{x\in p_{x^{\prime }}}\left(a_{x^{\prime }}I\left(x\right)+b_{x^{\prime }}-\tilde{J}\left(x\right)\right)=0$$

According to Equations (17) and (18), the estimation of variables $a_{x^{\prime }},b_{x^{\prime }}$ were:

(19) $$a_{x^{\prime }}=\frac{\frac{1}{N}\sum_{x\in p_{x^{\prime }}}I\left(x\right)\cdot \tilde{J}\left(x\right)-\frac{b_{x^{\prime }}}{N}\sum_{x\in p_{x^{\prime }}}I\left(x\right)}{\frac{\lambda }{\Gamma_{x^{\prime }}\left(x\right)}+\frac{1}{N}\sum_{x\in p_{x^{\prime }}}I\left(x\right)\cdot \tilde{J}\left(x\right)}$$

(20) $$b_{x^{\prime }}=\frac{\sum_{x\in p_{x^{\prime }}}\tilde{J}\left(x\right)-a_{x^{\prime }}\sum_{x\in p_{x^{\prime }}}I\left(x\right)}{N}$$

Applying Equation (20) to Equation (21), we have:

(21) $$a_{x^{\prime }}=\frac{\frac{1}{N}\sum_{x\in p_{x^{\prime }}}I\left(x\right)\cdot \tilde{J}\left(x\right)-\frac{1}{N}\sum_{x\in p_{x^{\prime }}}I\left(x\right)\cdot \frac{1}{N}\sum_{x\in p_{x^{\prime }}}\tilde{J}\left(x\right)}{\frac{\lambda }{\Gamma_{x^{\prime }}\left(x\right)}+\frac{1}{N}\sum_{x\in p_{x^{\prime }}}I\left(x\right)\cdot \tilde{J}\left(x\right)-\frac{1}{N}\sum_{x\in p_{x^{\prime }}}I\left(x\right)\cdot \frac{1}{N}\sum_{x\in p_{x^{\prime }}}\tilde{J}\left(x\right)}$$

(22) $$b_{x^{\prime }}=\frac{1}{N}\left(\sum_{x\in p_{x^{\prime }}}\tilde{J}\left(x\right)-a_{x^{\prime }}\sum_{x\in p_{x^{\prime }}}I\left(x\right)\right)$$

As mentioned above, transmission map *t*(*x*) and atmospheric light *A*(*x*) both satisfied the feature of local consistent. Thus, once the values of $a_{x^{\prime }},b_{x^{\prime }}$ were available, it can be derived that

(23) $$t\left(x\right)=\frac{1}{{\bar{a}}_{x}},\;A\left(x\right)=\frac{{\bar{b}}_{x}}{1-{\bar{a}}_{x}}$$

where ${\bar{a}}_{x}=\frac{1}{N}\sum_{x^{\prime }\in p_{x}}a_{x^{\prime }},\;{\bar{b}}_{x}=\frac{1}{N}\sum_{x^{\prime }\in p_{x}}b_{x^{\prime }}$. *N* is the pixel number in patch *p_x_* centered at *x*. ${\bar{a}}_{x}$ and ${\bar{b}}_{x}$ present the mean values of $a_{x^{\prime }}$ and $b_{x^{\prime }}$ in patch *p*_x_.

At this point, the values of the atmospheric light *A*(*x*) and the medium transmission map *t*(*x*) became available, hence, the dehazed image *J*(*x*) could be recovered easily according to Equation (3). As the recovered scene radiance *J*(*x*) will suffer from noise when the transmission *t*(*x*) is close to zero, we restricted the value of *t*(*x*) by a lower bound *t*_0_, which was experimentally fixed to 0.1 in Reference [16]. The final function used for restoring dehazed image *J*(*x*) can be expressed by:

(24) $$J\left(x\right)=\frac{1}{max\left(t\left(x\right),t_{0}\right)}\left(I\left(x\right)-A\left(x\right)\right)+A\left(x\right)$$

### 4.2. Label Candidates and Initialization

As mentioned above, the energy minimization process presented in Equation (15) requires an initial estimate $\tilde{J}\left(x\right)$ to start the operation. Theoretically, the choice of initial solution determines the optimization speed, thus we needed to select a close approximate to the solution as an initial estimate to speed up the process. The initial value $\tilde{J}\left(x\right)$ cannot be accessed directly, therefore, to obtain the initial value of dehazed image $\tilde{J}\left(x\right)$, we approximately estimated it through the coarse atmospheric light and transmission map with Equation (3).

**Figure 4:**
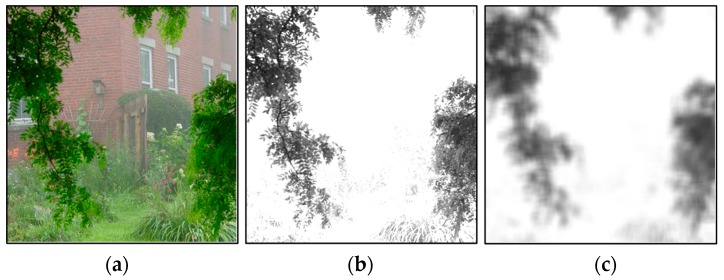
Initial value of the atmospheric light. (**a**) Input hazy image; (**b**) The value of Y(x); and (**c**) the blurred Y(x), namely the initial value of atmospheric light.

In this paper, we used the blurred Y(x) of the YIQ model [13] as the coarse value of atmospheric light $\tilde{A}\left(x\right)$. The value Y(x) and the blurred Y(x), the atmospheric light, are shown in Figure 4.

With Equation (3), the transmission map can be derived from:

(25) ln(A(x)−I(x))=ln(A(x)−J(x))+lnt(x)

and Equation (25) can be simplified as:

(26) $$\tilde{I}\left(x\right)=\tilde{J}\left(x\right)+\tilde{T}\left(x\right)$$

We estimated the initial transmission map values as the largest possible transmission map at each pixel. The observed image contained three color channels, and a single transmission map can be obtained to estimate each channel c∈{r,g,b}. Thus, the largest possible transmission map value occurs when $\tilde{J}\left(x\right)=0$, and the corresponding transmission map estimate ${\tilde{T}}_{c}\left(x\right)$ is:

(27) $${\tilde{T}}_{c}\left(x\right)={\tilde{Ι}}_{c}\left(x\right)$$

where *c* is a specific color channel. Next, we noted that there was only one map estimate in the three transmission map estimates ${\tilde{T}}_{c}\left(x\right),c\in \left\{r,g,b\right\}$ is the possible value for all three-color channels. Thus, we set the initial transmission map estimate $\tilde{t}\left(x\right)$ to the closest one over all channels:

(28) $$\tilde{t}\left(x\right)=e^{\underset{c\in \left\{r,g,b\right\}}{max}{\tilde{T}}_{c}\left(x\right)}=e^{\underset{c\in \left\{r,g,b\right\}}{max}{\tilde{I}}_{c}\left(x\right)}$$

In other words, the largest transmission map $\tilde{t}\left(x\right)$ was the valid map for all three-color channels. Note that when we maximized the ${\tilde{I}}_{c}\left(x\right)$, we determined A(x) by picking image points corresponding to direct observations of the sky or the brightest scene points in Reference [12].

**Figure 5:**
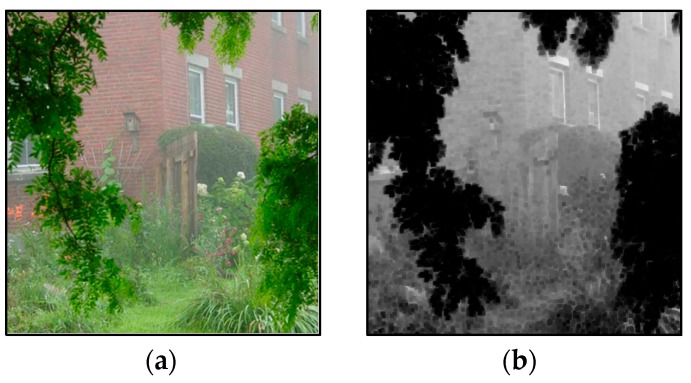
Initial value of the transmission map. (**a**) Input hazy image; and (**b**) the initial value of the transmission map.

Figure 5 shows the initial transmission map estimate computed from the hazy image, and illustrates the initial estimate capturing the scene transmission map structure well.

Recently, He [16] proposed an estimation of the transmission map based on the dark channel prior (DCP). The DCP is used as the initial estimate for the soft matting that imposes conventional smoothing on the transmission map estimation. Furthermore, the estimate transmission map is computed in local leading to halo artifacts. The transmission map in this paper was also estimated in local areas with the edge-preserving terms in the proposed local consistent MRF cost function, and our method finally resulted in a finer estimation.

Above all, we obtained the estimated value of the dehazed image $\tilde{J}\left(x\right)$ by applying the initial atmospheric light $\tilde{A}\left(x\right)$ and transmission map $\tilde{t}\left(x\right)$ to Equation (3). The redundant details in the atmospheric light and the transmission map were refined and correctly represented in Equation (16) during the joint minimization.

## 5. Experimental Results

To verify the effectiveness of the proposed dehazing method, we compared it with other MRF-based and soft-matting-based methods, including Tan [13], Nishino [14], Fattal [12] and He [16] on various hazy images. The experimental results in this paper consist of two parts. Part A discusses the dehazing results through a qualitative comparison of hazy images captured by CMOS sensor devices in the real-world. Part B presents the quantitative comparison of the respective dehazing results of the proposed method with other methods on real-world hazy images and synthetic hazy images. In addition, all the tests were implemented in the Matlab R2014a environment on an Intel Core (T) i7-3770 CPU @3.40 GHz processor with 8.00 GB of RAM running a Windows 7 operation system.

### 5.1. Qualitative Comparison among Real-World Hazy Images

We have stated that the local consistent MRF plays an important role for edge preservation. The edge-preserving term helps to recover better results. Most of the dehazing algorithms are able to obtain really good results in many hazy images. We used the dehazed images generated by different methods and their corresponding visible edges images to contrast the results markedly.

**Figure 6:**
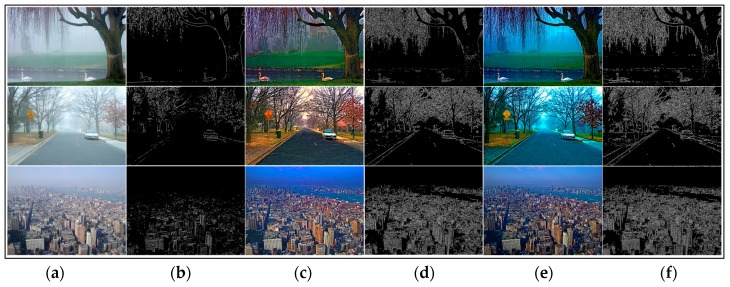
Comparison of our method to the method discussed by Tan [13] using real-world images. (**a**,**b**) are input hazy images and their corresponding visible edges, respectively; (**c**–**f**) are the dehazing results with corresponding visible edges generated by the method of Tan [13] and ours, respectively.

**Figure 7:**
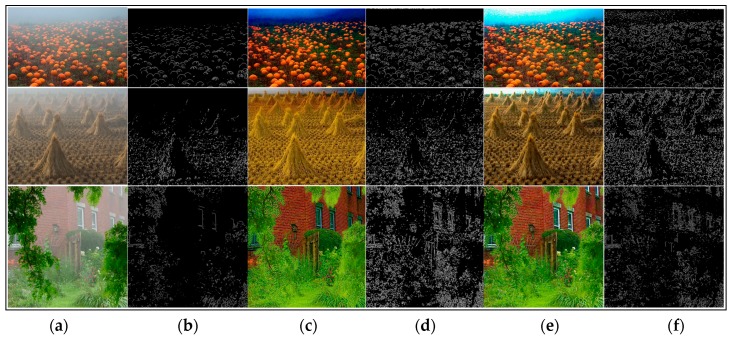
Comparison of our method to the method by Nishino [14] using real-world images. (**a**,**b**) input hazy images and their corresponding visible edges, respectively; (**c**–**f**) are the dehazing results with corresponding visible edges generated by the method of Nishino [14] and ours, respectively.

**Figure 8:**
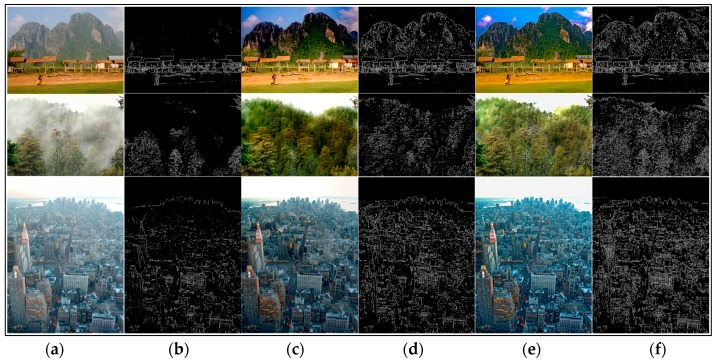
Comparison of our method to the method by Fattal [12] using real-world images. (**a**,**b**) are input hazy images and their corresponding visible edges, respectively; (**c**–**f**) are the dehazing results with corresponding visible edges generated by the method of Fattal [12] and ours, respectively.

**Figure 9:**
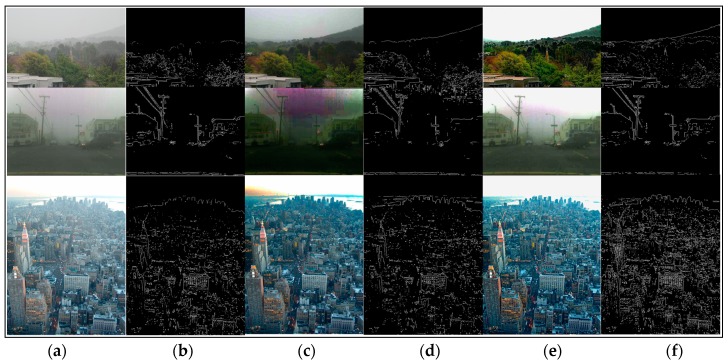
Comparison of our method to the He [16] soft matting based method using real-world images. (**a**,**b**) are input hazy images and their corresponding visible edges, respectively; (**c**–**f**) are the dehazing results with corresponding visible edges generated by the method of He [16] and ours, respectively.

As shown in Figure 6, Figure 7, Figure 8 and Figure 9, a qualitative comparison of results with the four state-of-the-art dehazing algorithms mentioned in References [12,13,14,16] were measured on different real-world hazy images. Among them, Figure 6, Figure 7 and Figure 8 show the results of our method when compared with the MRF-based methods. Figure 9 shows the comparison results between our method and soft-matting.

As shown in Figure 6, most haze was removed in Tan’s [13] results, and the scene details were restored. However, the results suffered significantly from over-enhancement and did not fully recover the scene colors. For instance, the road region of the second image was dark and the swans in the first image became brown. This was because Tan’s algorithm was based on maximizing the local contrast of the restored image and had the inherent problem of overestimating the dehazed image. The results of Nishino [14] had a similar problem which tended to introduce color distortion in the dehazed image. As observed in Figure 6c, the restored images were over-saturated and distorted, especially in the first image where the sky color was turned to a darker blue and the wheat was not its real color. Furthermore, Nishino’s algorithm also over-enhanced the local contrast. In some results (the third image in Figure 7), Nishino’s results and its corresponding visible edges showed more details than our algorithm, while their visual pleasures were not as good as ours. In all cases, the results of Fattal [12] (shown in Figure 8) all showed color distortion, such as the darker color of the wheat bundles around the mountain in the first image, the brighter green in the middle forest of the second image, and in the third image, the grayish tint to the buildings in the middle and far distance. In contrast, our results were natural and remaining in colors across the entire image. Regarding detail contrast, the results by Fattal [12] lacked details in the far scene, while our algorithm recovered a dehazed image with finer granularity even in areas such as the mountain at the top region of our recovered image in Figure 8f.

In Figure 6, Figure 7 and Figure 8, all of the algorithms compared did not produce halo artifacts. It was clearly observed that our results were slightly sharper than the dehazed images by other haze removal methods, given the main contribution of the edge-preserving term in our local consistent MRF model.

Figure 9 shows the comparisons between the proposed method and soft-matting. Our results were more saturated and have no apparent haze degradation. Observing this comparison, the algorithm in Reference [16] may exhibit halo artifacts near some edges, for instance, the mountain and the plant leaves in the first image. Additionally, the blocking effect existed obviously in the sky of the first and second images. Moreover, He’s method also introduced color distortion in the regions with white objects such as the sky regions in the second and third images where the reason was that the scene brightness was similar to the atmospheric light as the method recovering the transmission map used in Reference [16] ( based on the DCP) was invalid. In addition, the atmospheric light is also an important factor for estimating the transmission in [16]. Therefore, to obtain the correct transmission map, we required an accurate estimation of the atmospheric light. However, the estimating method for atmospheric light in He’s algorithm was the approximate, not its accurate value. Hence, He’s method has its limitations. In contrast, with close initial estimations, our method estimated the atmospheric light and transmission map jointly in the framework of local consistent MRF, and also with the terms of edge-preserving, so our method could avoid the halo artifacts and recovered most of the scene details in the dehazed image.

### 5.2. Quantitative Comparison

The real-world hazy images dehazing results were compared by entropy and Peak Signal-to-Noise Ratio (PSNR). Entropy describes the image information. PSNR is used to characterize the full degree of the image’s information as well as structure. Note that the higher PSNR and entropy scores imply more excellent restored quality. In Table 1, the PSNR and entropy comparison of Figure 6, Figure 7, Figure 8 and Figure 9 are provided. As seen, our results mostly achieved a higher PSNR and entropy than the other methods. In Table 2, the execution time comparison of Figure 6, Figure 7, Figure 8 and Figure 9 are provided. The experimental results show that our algorithm is slower than that of Tan [13], Nishino [14] and Fattal [12] due to local consistent block, but our algorithm avoids the use of high order energy terms, so that is faster than He [16].

**Figure 10:**
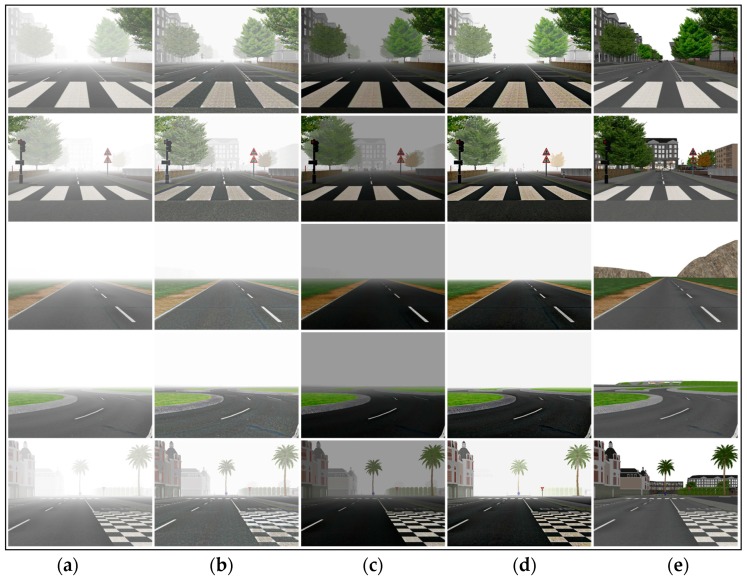
A comparison of the results of synthetic hazy images. (**a**) Synthetic hazy images; (**b**) Caraffa’s [15] results; (**c**) He’s [16] results; (**d**) Our results; and (**e**) ground truth images.

**Figure 11:**
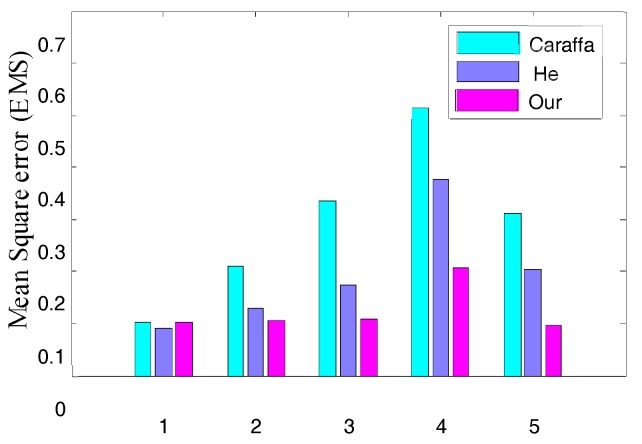
Mean Square Error (MSE).

**Figure 12:**
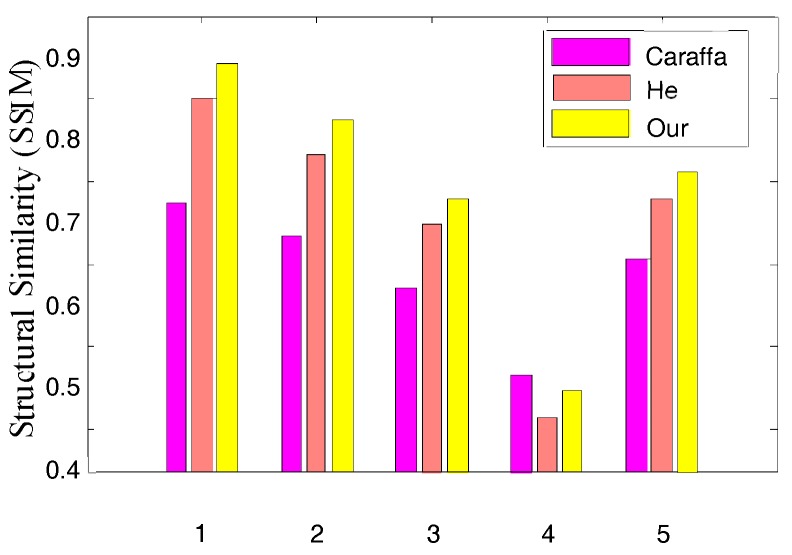
Structural Similarity Index of different algorithms. Structural Similarity Index Measurement (SSIM) of different algorithms.

To evaluate the proposed algorithm comprehensively, we used synthetic hazy images with their corresponding known haze-free images to quantify the dehazed images on various dehazing algorithms. Figure 10 shows the comparison on synthetic hazy images, and Figure 11 and Figure 12 show the Mean Square Error (MSE) and Structural Similarity Index Measurement (SSIM) values produced by different algorithms in Figure 10, respectively.

Lower MSE implies a greater similarity between the dehazed image and the referenced ground-truth image. From Figure 11, our algorithm had the lowest value in almost all cases, which meant that our results were closer to the ground-truth images and the dehazing effects more natural.

SSIM evaluates the ability to preserve the structural information of the algorithms where a higher SSIM implies a greater similarity between the dehazed image and the ground-truth image. Figure 12 shows the SSIM comparison results in Figure 10 where it was obvious that the proposed algorithm could preserve the structures.

In Table 3, the execution time comparison of Figure 10 are provided. The experimental results show that our algorithm is slower than that of Caraffa [15] due to local consistent block, but our algorithm avoids the use of high order energy terms, so that is faster than He [16].

## 6. Conclusions

Due to its small volume and low cost, the CMOS image sensor has attracted increased interest in the last few years and is expected to play a major role in outdoor surveillance, intelligent navigation, object tracking, and so on. Unfortunately, in many cases, the images captured by CMOS are often of low clarity under the effect of fog and haze weather. To ensure the quality of the image captured is more suitable for analysis in various surveillance applications, we proposed a novel framework for a novel local consistent MRF model for robust dehazing based on two clues where both the atmospheric light and the transmission map satisfy the features of local consistency. Accordingly, we created a linear model for the dehazed image with a local consistent transmission map and atmospheric light, and constructed a cost function in local consistent MRF. Interestingly, we found that the cost function had a dramatic relationship with the soft-matting; however, unlike soft-matting, the cost function had an additional edge-preserving term which resulted in the avoidance of halo artifacts and restoring more details. Consequently, the local consistent MRF model proposed could effectively produce haze-free restoration results. Qualitative and quantitative results demonstrated that the proposed local consistent MRF model performed better in hazy image restoration on both real-world images and synthetic data. Nevertheless, the proposed model had some limitations, such as reduced execution time and local artifacts without considering the semantic information of the whole image. Both problems will be studied in our future work.

## Figures and Tables

**Table 1:** Comparison of entropy and Peak Signal-to-Noise Ratio (PSNR) on the real-world image for each algorithm compared.

Image		Entropy			PSNR	
		Input	Tan	Ours	Tan	Ours
Figure 6	Image (1)	7.0079	7.2235	7.6043	9.9862	10.9585
	Image (2)	7.0079	7.3420	7.0079	9.3524	9.7479
	Image (3)	7.0955	7.6129	7.7114	10.6062	12.5667
**Image**		**Input**	**Nishino**	**Ours**	**Nishino**	**Ours**
Figure 7	Image (1)	7.1143	7.6770	7.7742	10.1835	16.3444
	Image (2)	7.1578	6.9343	7.2157	15.8571	15.9809
	Image (3)	6.5114	7.0954	7.4754	12.1857	14.7134
**Image**		**Input**	**Fattal**	**Ours**	**Fattal**	**Ours**
Figure 8	Image (1)	7.0878	7.3270	7.4739	12.6795	15.2639
	Image (2)	6.7272	6.9164	7.6595	9.3204	12.5983
	Image (3)	7.1773	7.2793	7.1832	16.1457	17.5572
**Image**		**Input**	**He**	**Ours**	**He**	**Ours**
Figure 9	Image (1)	5.6610	6.8479	7.1597	14.4612	12.5766
	Image (2)	6.4788	6.9625	6.9819	10.6480	18.4398
	Image (3)	7.1773	7.2793	7.1832	16.1457	17.5572

**Table 2:** Comparisons of arithmetic effectivity on the real-world image for running times (second) of the haze removal algorithm.

**Image**		**Size**	**Tan**	**Ours**
Figure 6	Image (1)	624 × 416	21.9913	30.5733
	Image (2)	596 × 396	18.4131	25.8991
	Image (3)	1024 × 768	53.9185	71.4230
**Image**		**Size**	**Nishino**	**Ours**
Figure 7	Image (1)	600 × 400	29.7421	33.8257
	Image (2)	465 × 384	21.8739	26.1618
	Image (3)	440 × 448	25.8193	29.8271
**Image**		**Size**	**Fattal**	**Ours**
Figure 8	Image (1)	512 × 348	18.9123	25.2018
	Image (2)	351 × 244	8.0125	12.9139
	Image (3)	576 × 768	36.9777	43.5120
**Image**		**Size**	**He**	**Ours**
Figure 9	Image (1)	660 × 440	107.9991	39.8194
	Image (2)	480 × 360	84.3372	23.7916
	Image (3)	576 × 768	181.0391	43.5120

**Table 3:** Comparisons of arithmetic effectivity in Figure 10/s.

Input	Size	Caraffa	He	Ours
Figure 10 Image (1)	640 × 480	36.8444	102.3404	42.4456
Figure 10 Image (2)	1376 × 1032	113.3376	387.5691	125.9364
Figure 10 Image (3)	512 × 384	18.9124	59.4183	24.7468
Figure 10 Image (4)	960 × 720	57.1672	197.8565	69.8188
Figure 10 Image (5)	736 × 552	42.3516	173.0013	58.8756
